# Chlamydia Peritonitis and Ascites Mimicking Ovarian Cancer

**DOI:** 10.1155/2016/8547173

**Published:** 2016-09-25

**Authors:** Anar Gojayev, Diana P. English, Matthew Macer, Masoud Azodi

**Affiliations:** ^1^Department of Obstetrics and Gynecology, Jamaica Hospital Medical Center, Jamaica, NY 11418, USA; ^2^Department of Obstetrics and Gynecology, Division of Gynecologic Oncology, Stanford University Hospital and Clinics, Stanford, CA 94305, USA; ^3^Department of Obstetrics and Gynecology, Division of Reproductive Endocrinology and Infertility, UCLA, Los Angeles, CA 94305, USA; ^4^Department of Obstetrics, Gynecology & Reproductive Sciences, Yale University School of Medicine, New Haven, CT 06510, USA

## Abstract

*Background*. Pelvic inflammatory disease (PID) rarely results in diffuse ascites. Severe adhesive disease secondary to PID may lead to the formation of inclusion cysts and even pelvic peritoneal nodularity due to postinflammatory scarring and cause an elevation of serum CA-125 levels. The constellation of these findings may mimic an ovarian neoplasm.* Case*. We report a case of a 22-year-old female who presented with multiple pelvic cysts and diffuse ascites due to* Chlamydia trachomatis* infection. The initial gynecologic exam did not reveal obvious evidence of PID; however, a positive* Chlamydia trachomatis* test, pathologic findings, and the exclusion of other etiologies facilitated the diagnosis.* Conclusion*.* Chlamydia trachomatis* and other infectious agents should be considered in the differential diagnosis of a young sexually active female with abdominal pain, ascites, and pelvic cystic masses. Thorough workup in such a population may reduce the number of more invasive procedures as well as unnecessary repeat surgical procedures.

## 1. Introduction

*Chlamydia trachomatis* infections may occur in multiple organs including lungs, lymph nodes, peritoneal cavity, and genitourinary systems. The overall increase in multipartner sexual activity among younger women has led to increased sexually transmitted infections as well as their acute and chronic sequelae. Consequently, intra-abdominal inflammatory disease resulting in ascitic peritonitis may also become a more common manifestation of* Chlamydia trachomatis* infection. Severe PID due to* Chlamydia trachomatis* may result in significant ascites, pelvic masses, and cul-de-sac nodularity on exam as well as an elevated Ca-125 tumor marker and imaging findings that mimic an ovarian malignancy.

## 2. Case

A 22-year-old G2 P-0-0-2-0 Hispanic patient was transferred from outside hospital for evaluation and management of worsening abdominal pain and findings of bilateral cystic adnexal lesions with septations and large ascites. Her obstetric and gynecologic history was significant for two dilatation and curettage procedures for elective abortion. Her surgical history was otherwise not significant. She was currently using oral contraceptive pills and continued to be sexually active. She denied any history of sexually transmitted diseases and did not have a recent pap smear. Her medical history was remarkable for bipolar disorder and depression. Her social history was notable for her being adopted and she did not know her biologic family. She also was a smoker.

She had previously presented to an emergency department (ED) at an outside hospital for evaluation of right lower quadrant pain without report of fever, chills, or abnormal vaginal discharge. She had no prominent gastrointestinal or urinary symptoms. Her urine pregnancy test was negative. After workup including a pelvic ultrasound, this pain was thought to be due to a ruptured ovarian cyst and was managed with analgesic medications. She however returned to the outside ED 2 weeks later with persistent pain and on repeat pelvic ultrasound was found to have larger bilateral septated ovarian cysts measuring up to 5 cm in largest dimension with free fluid seen in pelvis. She was transferred to our hospital for additional workup and management.

On presentation, she reported that the pain has moved from the right lower quadrant to the left lower quadrant and was aggravated by exercise. She also reported early satiety and noticed 15 pounds of unintentional weight gain in last 2 months.

Her examination revealed that she was afebrile and was hemodynamically stable. Her abdomen was soft but distended and tympanic in upper quadrants with mild tenderness in epigastrium on deep palpation. There was no rebound or guarding and a negative murphy's sign. A fluid wave was elicited at this examination. Pelvic exam revealed minimal yellow-white vaginal discharge, bilateral adnexal tenderness left more than right with pelvic fullness. The rectal exam was unremarkable.

Her blood test results revealed mild anemia with a hemoglobin of 10.8 g/dL. White blood cell and platelet count were within normal limits at 6,800/microliter and 358,000/microliter, respectively. The comprehensive metabolic profile was within normal limits and there was no evidence of liver dysfunction. Serum CA 125 and inhibin analysis demonstrated increased levels at 97.1 U/mL and 35 pg/mL, respectively. Serum concentrations of the other tumor markers were within normal limits.

*Chlamydia trachomatis* and* N. gonorrhoeae *DNA probe test were performed in the ED at the time of the vaginal exam.* C. trachomatis* test was positive and treatment was immediately initiated with 2 grams of single dose of intravenous azithromycin during her ED visit. Her partner was also treated. Since* C. trachomatis* testing was positive, other serological tests were performed to rule out sexually transmitted infections (STI) such as HIV and VDRL with the patient's consent. However, all of the subsequent STI testing was negative.

Computed tomography (CT) scan of the abdomen and pelvis had been ordered due to the ultrasound findings and revealed rim enhancing complex cystic lesions in the right and left adnexa measuring 3.5 cm and 1.4 cm, respectively, with multiple other nodules in both adnexa (Figure 4). Enhancing soft tissue densities in the mesentery, peritoneal enhancement in the pelvis, a large amount of ascites, and an indeterminate 1 cm liver lesion was also appreciated on CT scan (Figure 3). Differential diagnoses at this point were broad, including infectious, inflammatory, or possible malignant diseases. Chest imaging was noncontributory. Considering the CT scan findings of mesenteric disease and liver lesion, which could very well be seen in metastatic ovarian cancers, we decided to further investigate for the possibility of a malignancy.

Workup was initiated with a paracentesis which was diagnostic and therapeutic at this point. Peritoneal fluid appeared straw-colored, cytologic examination was negative for malignant cells, and no fungal organism or bacteria were detected with either the direct microscopic examination of the fluid, gram stain, or culture.

**Figure 1:**
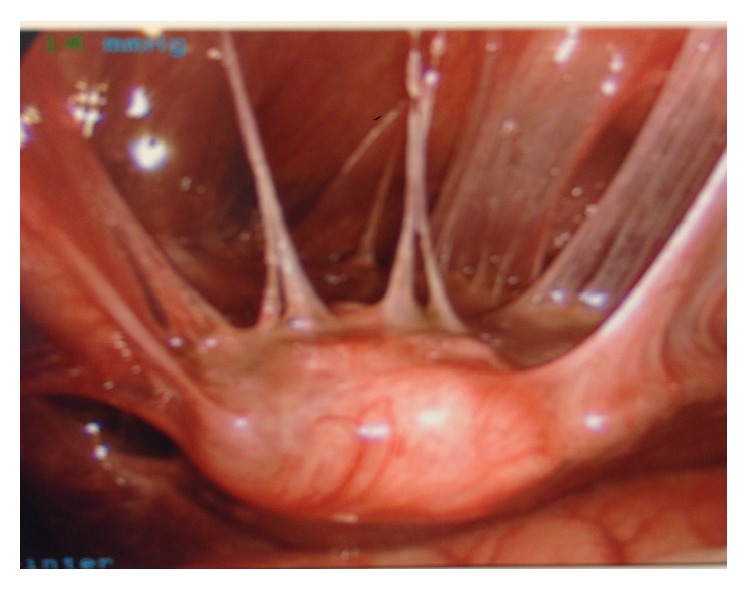
Laparoscopic image showing severe pelvic fibrous adhesions surrounding the uterus and from the uterus to anterior abdominal wall. Uterus appears to be suspended by these adhesions.

**Figure 2:**
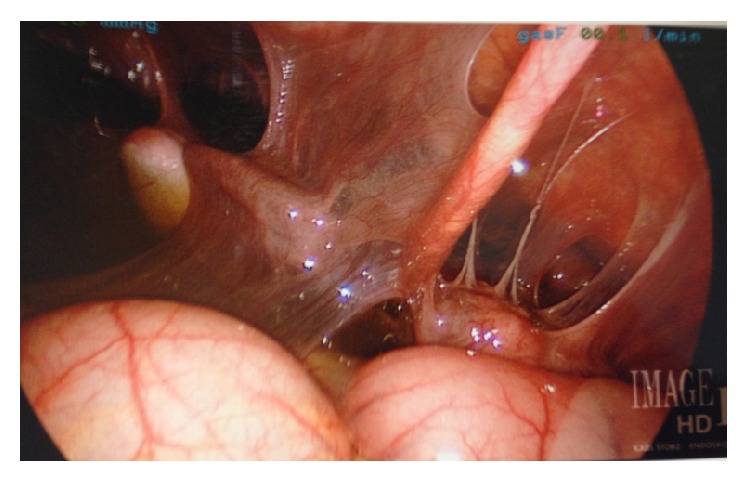
Laparoscopic image showing severe perihepatic fibrous adhesions.

**Figure 3:**
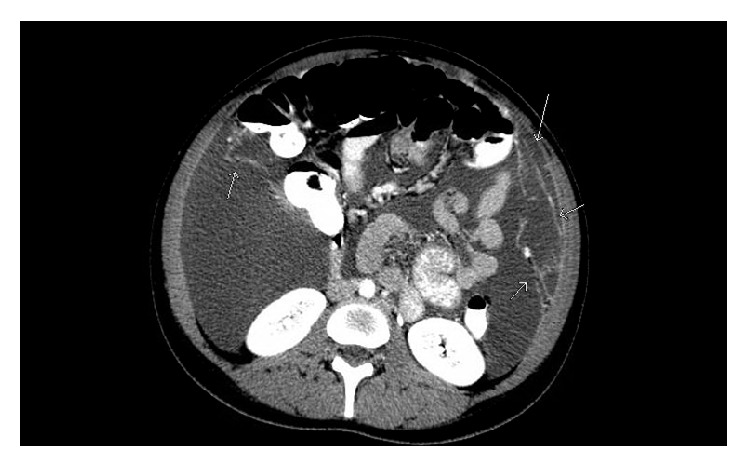
CT scan of the abdomen and pelvis revealing a significant amount of ascites and enhancing predominantly linear soft tissue densities in the right and left upper quadrant labelled by arrows.

**Figure 4:**
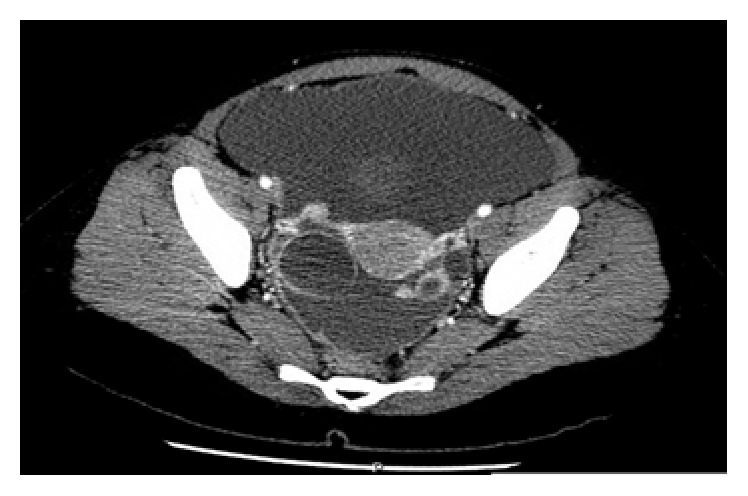
CT scan showing rim enhancing cystic lesions in the right and left adnexa measuring 3.5 cm and 1.4 cm, respectively, with multiple other nodules in both adnexa, peritoneal thickening, and abdominopelvic ascites.

Given the limited knowledge of her family history as well as the possibility of a germ cell ovarian tumor in this age group, the patient was then counseled regarding the option of a diagnostic laparoscopy for further investigation of the findings. She consented to an exploratory laparoscopy, possible unilateral versus bilateral cystectomies, liver biopsy, and any other necessary procedures. Intraoperative findings were consistent with Fitz-Hugh-Curtis syndrome, loculated ascites, and overall severe PID (Figures 1 and 2). She ultimately underwent segment 3 liver wedge resection for 1 cm nodule, pelvic washings, omental and peritoneal biopsies, and extensive lysis of abdominopelvic adhesions. The liver lesion was a very unexpected finding even with* Chlamydia trachomatis* infections and the managing physicians wanted to rule out other etiologies such as a primary liver lesion versus a metastatic deposit from another site.

Pathologic examination demonstrated fibrous tissue on multiple peritoneal and pelvic nodule biopsies and the omental biopsy showed atypical lymphoid proliferation. The liver biopsy showed nonspecific reactive hepatitis.

Peritoneal fluid flow cytometry and B- and T-cell gene rearrangement tests were also performed due to the finding of atypical lymphoid proliferation on pathology. These results all returned without significant abnormalities and the atypical lymphoid proliferation was therefore thought to be as a result of severe PID. In strong support of this diagnosis was also the ascitic fluid* Chlamydia trachomatis* IgG antibody titer of 1 : 1024.

Therefore, this patient was diagnosed with a complicated PID. As she had previously been treated with IV azithromycin, she was then treated with metronidazole 500 mg twice daily and doxycycline 100 mg twice daily after receiving a single dose of ceftriaxone 250 mg intramuscularly. She had an uncomplicated postoperative course and was discharged home on postoperative day 3.

By postoperative day 25 she was again complaining of abdominal distension and was found to have a reaccumulation of ascites. A repeat therapeutic paracentesis was performed. She was again found to have mainly loculated ascites and as such 600 cc of serosanguineous peritoneal fluid was drained for symptomatic relief. She continued with the antibiotic therapy and received a total of 28 days of antibiotics after discharge. She was completely asymptomatic at a follow-up appointment eight months after her initial presentation. Her follow-up pelvic ultrasound showed normal appearing ovaries bilaterally and a minimal amount of pelvic free fluid.

## 3. Discussion

Diagnosis of* C. trachomatis* infection is commonly based on the detection of IgM and IgG antibodies to* C. trachomatis* in body fluids, such as secretions, tears, serum, and cervical mucus by immunofluorescence methods. The antibody titers in this patient's ascites supported the presumed diagnosis of* C. trachomatis* peritonitis with ascites formation. There are very few reports on determination of antibody titers in ascites [1]. The first reported cases of ascites associated with chlamydia were in 1978 [2]. It is thought based on the literature that the high antibody titers in the ascitic fluid are suggestive of* C. trachomatis* peritonitis. In addition other findings suggestive of* C. trachomatis* ascites include the absence of chronic liver disease with ascitic fluid analysis showing a predominance of lymphocytes and the finding of an exudative process as evidenced by high protein content [2, 3]. Examination of our patient's ascites also showed similar results.

The general management of* C. trachomatis* associated ascites is the administration of azithromycin or doxycycline. The review of the previously reported literature demonstrates that patient's symptoms including ascites formation generally decrease after treatment with antibiotics [4–6].

It has also been suggested that* C. trachomatis* ascites may be a self-limiting condition and the resolution may not be necessarily dependent on the administration of antibiotics [1, 7].

As the incidence of* C. trachomatis* genitourinary infections is on the rise, clinicians may encounter more cases of* Chlamydia trachomatis* peritonitis, ascites, and adhesion formation. Therefore,* C. trachomatis* and other infectious etiologies should be in the differential diagnosis for a sexually active premenopausal patient with abdominal pain, ascites, and pelvic cystic masses and appropriate testing should be pursued early in the workup of these patients. The main difficulty in cases as this one is to balance the consideration of a patient having more than one etiology for their clinical picture with the risk of over investigation. The managing team in this case had the aim of not missing a potential cancer diagnosis given the imaging findings despite the positive* Chlamydia trachomatis* nucleic acid probe.

This case is important as it is a rare presentation of* Chlamydia trachomatis* infection and underscores the relevance of standard workup for sexually transmitted infections combined with ascitic fluid microbiology analysis to evaluate young women with ascites and pelvic masses before proceeding with more invasive surgical procedures.

## Additional Points

(1)* C. trachomatis* infections may occur in multiple organs including lungs, lymph nodes, peritoneal cavity, and genitourinary systems. (2) Severe adhesive disease may lead to the formation of inclusion cysts in the pelvis that may mimic an ovarian neoplasm. (3) Patients with PID complicated by tuboovarian abscess formation, ascites, or nodularity due to postinflammatory scarring may mimic an ovarian neoplasm. (4)* C. trachomatis* and other infectious etiologies should be in the differential diagnosis in a young sexually active patient with abdominal pain, ascites, and pelvic cystic masses.

## References

[1] Yanagisawa N., Tomiyasu H., Hada T. (1992). Chlamydia trachomatis peritonitis: report of a patient presenting spontaneous regression of ascites. *Internal Medicine*.

[2] Mueller-Schoop J. W., Wang S. P., Munzinger J. (1978). *Chlamydia trachomatis* as possible cause of peritonitis and perihepatitis in young women. *British Medical Journal*.

[3] Ugianskiene A. (2013). Chlamydial infection with marked ascites that simulated ovarian cancer. *Ugeskrift for Laeger*.

[4] Haight J. B., Ockner S. A. (1988). Chlamydia trachomatis perihepatitis with ascites. *The American Journal of Gastroenterology*.

[5] Guagenti R. C., Berman A. L., Cohen N. N. (1989). Chlamydial ascites. *Digestive Diseases and Sciences*.

[6] Votte-Lambert A., Joly J. P., Becuwe C., Eb F., Capron J. P., Dupas J. L. (1990). *Chlamydia trachomatis* peritonitis: another cause of protein-rich lymphocytic ascites. *Journal of Clinical Gastroenterology*.

[7] Mori K., Tomizawa Y., Arai R. (2003). Fitz-Hugh-Curtis syndrome presenting spontaneous regression of ascites. *Nihon Naika Gakkai zasshi. The Journal of the Japanese Society of Internal Medicine*.

